# Telemedicine to Support Heart Failure Patients during Social Distancing: A Systematic Review

**DOI:** 10.5334/gh.1175

**Published:** 2022-12-19

**Authors:** Serlie Fatrin, Salwa Auliani, Samuel Pratama, Thiara Maharani Brunner, Bambang Budi Siswanto

**Affiliations:** 1 Faculty of Medicine, Universitas Indonesia, Jakarta, Indonesia; 2 Department of Cardiology and Vascular Medicine, National Cardiovascular Center Harapan Kita, Jakarta, Indonesia

**Keywords:** heart failure, telemedicine, mortality rate, quality of life, hospitalisation rate

## Abstract

**Background:**

Heart failure (HF) has been described as an emerging pandemic as its prevalence continues to rise with a growing and aging population. HF patients are more vulnerable to infections with higher risk of hospitalisation, morbidity, and mortality. During this COVID-19 pandemic, telemedicine has emerged as an alternative to usual out-patient care. This study aimed to systematically review available literature regarding the effect of telemedicine on mortality, health-related quality of life (HR-QoL), and hospitalisation rate of HF patients.

**Method:**

A literature search was conducted on five databases (PubMed, Medline, EMBASE, SCOPUS and Cochrane Central Database) up to 21^st^ May 2022. Data from studies that fulfilled the eligibility criteria were collected and extracted. Included studies were critically appraised using suitable tools and extracted data were synthesized qualitatively.

**Results:**

A total of 27 studies were included in the qualitative synthesis with a total of 21,006 patients and sufficient level of bias. Reduction in the mortality rate, HF-related hospitalisation rate, and improvement in the HR-QoL were shown in most of the studies, although only some were statistically significant.

**Conclusions:**

The use of telemedicine is a promising and beneficial method for HF patients to acquire adequate health care services. Further studies in this field are needed, especially in developing countries and with standardized method, to provide better services and protections for HF patients. Telemonitoring and patient-centred partnership via interactive communication between healthcare team and patients is central to successful telemedicine implementation.

**PROSPERO Registration Number:**

CRD42021271540.

## Introduction

Heart failure is a complex, debilitating syndrome characterized by symptoms of breathlessness, ankle swelling and fatigue that occurs with minimal physical exertion or even at rest. The heart performs its systolic and diastolic function with diminished capacity as a result of myocardium injury and hence, metabolic demands of the body cannot be fulfilled. The resulting fall in cardiac output leads to activation of neurohormonal responses, such as the sympathetic nervous system and renin angiotensin-aldosterone system (RAAS), to maintain cardiovascular homeostasis. In the long term, prolonged activation of the neurohormonal system exacerbates cardiac injury. It has been known to be responsible for HF progression that is associated with poor morbidities and mortalities [1,2]. Furthermore, as a chronic and progressive disease, HF results in higher rate of rehospitalisation and poor quality of life (QOL).

Heart failure has been described as an emerging pandemic as its prevalence continues to rise with a growing and aging population. The risk increases over 20-fold in people aged ≥ 60 years old [3]. It poses a great clinical, social and economic challenge, especially in low-to-middle income countries where its outcome is largely influenced by low health-care infrastructure availability, workforce shortages and poorer access of care and quality [4].

Nevertheless, with advancement of digital technologies, the use of telemedicine has scaled up as a strategy to help address workforce shortages and reach patients in rural or underserved areas. This issue is especially prominent in low- to middle-income countries such as Indonesia, where a large number of populations are widely diverse and dispersed across many islands. Furthermore, it has gained popularity due to the efforts of maintaining mandatory social distancing during the COVID-19 pandemic. Patients become reluctant to seek medical help due to fear of contracting the severe acute respiratory syndrome coronavirus 2 (SARS-CoV-2) infection [5]. Telemedicine becomes the safest medium for patients to interact with clinicians and attempts to minimize substantial delays in management of HF patients. It will serve to maintain communication between physicians and patients as well as delivery of information and education, which is key to a sustainable self-care for individuals with HF.

The European Society of Cardiology has recognized its potential with a low level of recommendation (IIb), and termed it as, ‘remote patient management’ [6]. Previous clinical studies suggest that telemedicine lowers mortality and morbidity rates, but results have been inconsistent due to various systems of telemedicine and study designs (durations and population demographics) [7]. Telemedicine can be carried out invasively or non-invasively. An invasive telemonitoring employs the use of implantable electronic cardiac devices (implantable cardioverter-defibrillator or cardiac resynchronization therapy) with activated home monitoring features [7]. Non-invasive telemedicine can be delivered via structured telephone call, virtual visits, use of mobile apps or wearable devices connected to software and built-in algorithm systems to detect signs of abnormalities. Therefore, this study aims to evaluate the outcome of telemedicine in heart failure patients’ mortality rate, hospital admission rate, and quality of life (QOL) compared to standard outpatient visits.

## Methods

### Protocol and Registration

This systematic review was conducted based on the Preferred Reporting Items for Systematic Review and Meta-Analysis (PRISMA) Statement. The protocol of this systematic review has been registered in The International Prospective Register of Systematic Review (PROSPERO) database (CRD42021271540).

### Eligibility Criteria

Eligible studies included systematic reviews and randomized controlled trials that aimed to investigate the use of telemedicine compared to standard of care in heart failure patients and their effects on patients’ mortality, hospitalisation rate and quality of life. Participants that were included were heart failure patients aged ≥ 45 years old and classified under New York Heart Association (NYHA) Functional Classification II-IV. We excluded studies with HF patients with the following etiologies: pregnancy-related, congenital heart disease, valvular heart disease and present embolism. Studies without sufficient data, such as missing information on patients’ baseline characteristics or incomplete results, were excluded.

### Search Strategy

Literature search was performed using PubMed, Medline, EMBASE, SCOPUS and Cochrane Central Database, along with manual hand searching using keywords listed in Table 1, up to 21 May 2022. Additional searching was performed manually through relevant bibliographies of selected systematic reviews and meta-analysis. Selection of studies was restricted to those published in English or Bahasa Indonesia, with full text availability and no time limit. Title and abstracts generated using the search terms were identified and screened using our eligibility criteria. Screening for eligible records was done independently by five investigators (SF, SA, SP, TMB). Decisions for inclusion and disputes were settled by discussions amongst investigators with the help of a blinded, independent reviewer [8]. Flow of study selection was presented according to the PRISMA flow chart of study selection.

**Table 1:** Search queries of this systematic review.


DATABASE	SEARCH QUERY	INITIAL HITS

PubMed	(((((HFrEF) OR (heart failure)) OR (congestive heart failure)) AND (((((((((telemedicine) OR (online follow up)) OR (telecardiology)) OR (ehealth)) OR (e-health)) OR (online consultation)) OR (telehealth)) OR (virtual care)) OR (telemonitoring))) AND (((outpatient visitation) OR (offline follow up)) OR (in person appointment))) AND (((((((((Minnesota living with heart failure questionnaire) OR (quality of life)) OR (hospitalisation rate)) OR (hospitalisation)) OR (inpatient admission)) OR (admission rate)) OR (mortality)) OR (death)) OR (survival rate))	68

Medline	(((Heart Failure or CHF or HF or Congestive Heart Failure).mp. [mp = title, abstract, original title, name of substance word, subject heading word, floating sub-heading word, keyword heading word, organism supplementary concept word, protocol supplementary concept word, rare disease supplementary concept word, unique identifier, synonyms]) OR (Heart Failure.mp. or exp Heart Failure/) OR (HFrEF.mp.) OR (Congestive Heart Failure.mp.)) AND (((Online follow-up or telemedicine or online consultation or eHealth or telehealth or e-Health or telecardiology).mp. [mp = title, abstract, original title, name of substance word, subject heading word, floating sub-heading word, keyword heading word, organism supplementary concept word, protocol supplementary concept word, rare disease supplementary concept word, unique identifier, synonyms]) OR (Telemedicine.mp. or exp Telemedicine/)) AND (((Offline follow-up or Outpatient visitation or in-person appointment).mp. [mp = title, abstract, original title, name of substance word, subject heading word, floating sub-heading word, keyword heading word, organism supplementary concept word, protocol supplementary concept word, rare disease supplementary concept word, unique identifier, synonyms]) OR (Outpatient Visitation.mp. or exp Ambulatory Care/)) AND ((quality of life.mp. or exp “Quality of Life”/) OR (hospitalisation.mp. or exp hospitalisation/) OR (Admission Rate.mp. or exp Patient Admission/) OR (exp Mortality/or Mortality.mp.) OR (Survival rate.mp. or exp Survival Rate/) OR ((Quality of Life or QoL or hospitalisation or Inpatient admission or Admission Rate or hospitalisation rate or Mortality or Death or Survival rate).mp. [mp = title, abstract, original title, name of substance word, subject heading word, floating sub-heading word, keyword heading word, organism supplementary concept word, protocol supplementary concept word, rare disease supplementary concept word, unique identifier, synonyms]) OR (Death.mp. or exp Death/))	24

EMBASE	((heart failure OR congestive heart failure) AND (follow up OR online system OR telemedicine OR consultation OR teleconsultation OR telehealth OR telecardiology) AND (outpatient OR outpatient care OR outpatient department) AND (quality of life OR questionnaire OR hospitalisation OR hospital admission OR hospital mortality OR mortality rate OR mortality OR cardiovascular mortality OR survival rate))	180

SCOPUS	( TITLE-ABS-KEY ( ( heart AND failure ) ) OR TITLE-ABS-KEY ( ( hf ) ) OR TITLE-ABS-KEY ( ( congestive AND heart AND failure ) ) OR TITLE-ABS-KEY ( ( chf ) ) OR TITLE-ABS-KEY ( ( hfref ) ) AND TITLE-ABS-KEY ( ( telemedicine ) ) OR TITLE-ABS-KEY ( ( online AND follow-up ) ) OR TITLE-ABS-KEY ( ( online AND consultation ) ) OR TITLE-ABS-KEY ( ( ehealth ) ) OR TITLE-ABS-KEY ( ( telehealth ) ) OR TITLE-ABS-KEY ( ( e-health ) ) OR TITLE-ABS-KEY ( ( telecardiology ) ) OR TITLE-ABS-KEY ( ( virtual AND care ) ) OR TITLE-ABS-KEY ( ( telemonitoring ) ) AND TITLE-ABS-KEY ( ( outpatient AND visitation ) ) OR TITLE-ABS-KEY ( ( offline AND follow-up ) ) OR TITLE-ABS-KEY ( ( in AND person AND appointment ) ) AND TITLE-ABS-KEY ( ( minnesota AND living AND with AND heart AND failure AND questionnaire ) ) OR TITLE-ABS-KEY ( ( mlhfq ) ) OR TITLE-ABS-KEY ( ( quality AND of AND life ) ) OR TITLE-ABS-KEY ( ( qol ) ) OR TITLE-ABS-KEY ( ( hospitalisation ) ) OR TITLE-ABS-KEY ( ( inpatient AND admission ) ) OR TITLE-ABS-KEY ( ( admission AND rate ) ) OR TITLE-ABS-KEY ( ( mortality ) ) OR TITLE-ABS-KEY ( ( death ) ) OR TITLE-ABS-KEY ( ( survival AND rate ) )	3

Cochrane Central Database	#1 MeSH descriptor: [Heart Failure] explode all trees #2 “Heart Failure” OR HF OR “Congestive Heart Failure” OR CHF OR HFrEF #3 MeSH descriptor: [Telemedicine] explode all trees #4 Telemedicine OR “Online follow-up” OR “online consultation” OR eHealth OR telehealth OR e-Health OR telecardiology #5 MeSH descriptor: [Outpatients] explode all trees #6 Outpatient OR “Outpatient visitation” OR “offline follow-up” OR “in person appointment” #7 “Quality of life” OR QoL OR “Minnesota Living with Heart Failure Questionnaire” OR MLHFQ OR hospitalisation OR hospitalisation OR “inpatient admission” OR “admission rate” OR mortality OR death OR “survival rate” #8 #1 OR #2 #9 #3 OR #4 #10 #5 OR #6 #11 #7 AND #8 AND #9 AND #10	94


### Data extraction

Data extraction was performed by three reviewers (SF, SA, SP) and checked by three independent reviewers; any disputes regarding data extraction were discussed within the review team. Data were extracted to an excel spreadsheet in a standardized form, including study citations, baseline characteristics of participants, methods of intervention and study findings. Baseline data such as study settings, sample size, patient characteristics (age, gender, NYHA class, and left ventricle ejection fraction (LVEF), if available), types of intervention, as well as measures of effect and method of analysis were extracted from the included studies. Extracted outcomes were mortality rate, hospitalisation rate, as well as the quality of life of heart failure patients after receiving patient care via telemedicine compared to standard outpatient visit. Patients in the studies were followed for at least three months. The corresponding author of included studies with missing or incomplete data was contacted via email.

### Quality Assessment and Data Synthesis

Studies were assessed for risk of bias using the A MeaSurement Tool to Assess systematic Reviews 2 (AMSTAR 2) and Physiotherapy Evidence Database (PEDro) scale appraisal tools by five independent reviewers (SF, SGA, SP, TMB, BBS) for systematic reviews and RCTs, respectively. The AMSTAR 2 rating overall confidence was concluded as high if there was no to one non-critical weakness, as moderate if there was more than one non-critical weaknesses, as low if there was one critical weakness without non-critical weakness, and as critically low if there were more than one critical weaknesses [9]. The summary of assessment using PEDro scale was ‘excellent’ for scores of 9–10, ‘good’ for scores of 6–8, ‘fair’ for scores of 4–5, and ‘poor’ for scores of 0–3 [10].

We performed a qualitative analysis of the included studies and any discrepancies were resolved by discussion amongst reviewers with the help of an independent reviewer until a conclusion was reached. Data synthesis consisted of the study size, method of analysis used in each study, the appropriate measures of effect (odds ratio, hazard ratio, relative risks) and its confidence interval, as well as the risk of bias. Quality assessment of included studies was also tabulated.

## Results

### Study selection

**Figure 1:**
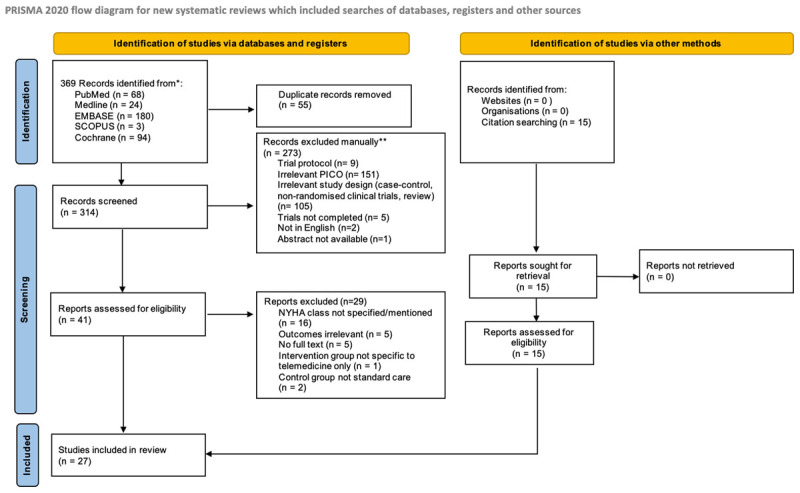
The PRISMA flow diagram for the systematic review.

Literature search using the listed keywords across five databases yielded 369 papers. After duplicates were removed, titles and abstracts of 314 records were screened. A total of 273 records were removed due to irrelevant titles and abstracts. Forty-one full-text articles were assessed for eligibility, 29 of which were excluded for reasons stated below. An additional 15 studies were found manually from bibliographies of relevant papers. A total of 27 studies were included in the qualitative synthesis. Figure 1 represents the PRISMA Statement to illustrate the flow of our study selection.

### Study Characteristics and Critical Appraisal

The characteristics of included systematic reviews and RCTs are summarized in Table 2 and Table 3, respectively. The 27 included studies consisted of two systematic reviews and twenty-five RCTs with a total of 21,006 patients. The two systematic reviews had included studies that were done in various locations, including both developed and developing countries, with populations of mean age ranging from 45 to 78 years old. One systematic review employed the use of structured telephone support or non-invasive home telemonitoring [7]. Meanwhile, the other review used m-Health technology to focus on distributing health education to HF patients [11]. Assessment of risk of bias using AMSTAR 2 for both systematic reviews yielded a low level of confidence (see Table 5).

**Table 2:** Summary of study characteristics and population demographics of systematic reviews.


AUTHORS	YEAR PUBLI-SHED	STUDY TYPE	STUDY LOCATION	STUDY DURATION	SAMPLE CHARACTERISTICS					TYPES OF INTERVENTION

					MEAN/MEDIAN AGE (YEARS)	SAMPLE SIZE	MALE/FEMALE (%)	NYHA CLASS	LVEF (%)	

Inglis et al.	2015	Systematic review and meta-analysis	**STS:** USA 14), Australia (1), Argentina (1), Brazil (1), Canada (1), Germany (1), India (1), Iran (1), Italy (1) and two studies which were involved several European countries (Germany, Netherlands, UK, Poland, Italy). **HT:** Italy (3), USA (3), Canada (2), Austria (1), Belgium (1), Finland (1), France (1), Germany (1), Sweden (1), The Netherlands (1), UK (1) and two studies involved several European countries (Germany, The Netherlands and the UK; UK, Poland and Italy)	6 months	**STS** = 45–75 **HT** = 55–78	41 studies **STS** = 9332 **HT** = 3860	**STS** = 64/36 **HT** = 72/28	**STS** = II–III **HT** = III	N/A	Structured telephone support or non-invasive home telemonitoring compared with usual post-discharge care

Allida et al.	2020	Systematic review	Australia (1), China (1), Iran (1), Sweden (1), and The Netherlands (1)	1–12 months	60–75	5 studies 1010	63/37	II–III	N/A	mHealth-delivered education interventions


**Abbreviations:** NYHA, New York heart association; LVEF, left ventricle ejection fraction; USA, United States of America; UK, United Kingdom; STS, structured telephone support; HT, non-invasive home telemonitoring; PDA, personal digital assistant; RTM, remote telemedical management N/A, not available/not known/not mentioned.

**Table 3:** Summary of study characteristics and population demographics of randomised control trials.


AUTHORS	YEAR PUBLISHED	STUDY TYPE	STUDY LOCATION	STUDY DURATION	SAMPLE CHARACTERISTICS				

					MEAN/MEDIAN AGE (YEARS)	SAMPLE SIZE	MALE/FEMALE (%)	NYHA CLASS	LVEF (%)

Villani et al.	2014	RCT	Italy	12 months	72	81	**Integrated managemen**t 75/25 **Usual care:** 73/27	III–IV	Mean LVEF 32 (5)

Pedone et al.	2015	RCT	Italy	6 months	80	90	**Telemonitoring** 47/53 **Control** 30/70	II–IV	N/A

Kenealy et al.	2015	RCT	New Zealand	6 months	72	98	**Telecare** 61/39 **Usual care** 71/29	III–IV	N/A

Pekmezaris et al.	2019	RCT	USA	3 months	59.9	104	57/43	II–III	61% had reduced EF <40%

Riegel B et al.	2002	RCT	USA	6 months	72 (12)	358	**Telemedicine** 54/46 **Usual care** 47/59	II–IV	Mean LVEF 41.9 (17.0)

Benatar D et al.	2003	RCT	USA	12 months	63 (12)	216	**NTM** 64/36 **HNV** 38/62	III–IV	**NTM** 38.05 (13.70) vs **HNV** 38.83 (13.97)

Dar O et al.	2009	RCT	UK	6 months	72 (12)	182	**Telemonitoring** 68/34 **Usual care** 65/35	II–IV	Mean LVEF was not reported 39% had LVEF >40%

Koehler F et al.	2011	RCT	Germany	24 months	66.9 (10.8)	710	**RTM** 81/19 **Usual care** 82/18	II–III	Criteria LVEF <35% Mean LVEF 26.9 (5.7) vs 27.0 (5.9)

Jerant AF et al.	2001	RCT	USA	6 months	**Video-based telecare** 66.6 (10.9) **Group telephone** 71.3 (14.1) **Usual care** 72.7 (11.4)	37	**Video-based telecare** 46/54 **Group telephone** 42/58 **Usual care** 50/50	II–IV	N/A

Kurtz B et al.	2011	RCT	France	12 months	68 (11)	138	**Telemonitoring** 83/17 **HF clinic** 86/14 **Usual care** 75/25	II–IV	Criteria LVEF <45% Mean LVEF 32 (10) vs 30 (8) vs 32 (8)

Melin M et al.	2018	RCT	Sweden	6 months	75(8)	72	**Intervention group** 66/34 **Control group** 70/30	II–IV	Mean LVEF was not reported 36% had **HFpEF** 54% had **HFrEF**

Antonicelli R et al.	2008	RCT	Italy	12 months	78.2 (7.3)	57	**HT** 57/43 **CG** 66/34	II–IV	Criteria LVEF <40% Mean LVEF **HT** 35 (6) vs **CG** 37(7)

Baker DW et al.	2011	RCT	USA (4 different sites)	12 months	60.7(13.1)	605	**BEI** 52/48 **TTG** 52/48	II–IV	Mean LVEF was not reported, but 60% had LVEF <45%

Blum K et al.	2014	RCT	USA	48 months	**Monitor group** 73 ± 8 **Usual care** 72 ± 10	206	**MG** 70.30 **UC** 72/28	II–IV	Mean LVEF **MG** 29 ± 15 **UC** 29 ± 15

DeWalt DA et al.	2006	RCT	USA	12 months	**Intervention group** 63 ± 9 **Control group** 62 ± 11	123	**IG** 58/42 **CG** 55/45	II–IV	Criteria LVEF < 40% Mean LVEF 39% vs 44%

Ferrante D et al.	2010	RCT	Buenos Aires, Argentina	36 Months	**IG** (n = 760) 64.8 (13.9) **CG** (n = 758) 65.2 (12.7)	1,518	**IG** 72.6/27.4 **CG** 68.9/31.1	II–IV	Mean LVEF was not reported, but 80% had LVEF <40%

Goldberg LR et al.	2003	RCT	Multiple centres in United States	6 Months	**Intervention group** (n = 138) 57.9 (15.7) **Standard care** (n = 142)60.2 (14.9)	280	**IG** 69.6/30.4 **CG** 65.5/34.5	III–IV	Criteria LVEF <35% **IG** 21.6 (6.8) **CG** 21.8 (6.8)

Mizukawa M et al.	2019	RCT	Hiroshima, Japan	24 Months	**Usual Care** 74.5 (12.1) **Self-Management** 69.4 (12.9) **Collaborative management** 70.5(13.3)	60	**Usual Care** 52.6/48.4 **Self-Management** Male (n = 15) 83.3/16.7 **Collaborative management** 50/50	III–IV	Criteria LVEF <40% or >40% **Usual Care** 42.1 (16.5) **Self-Management** 42 (14.7) **Collaborative management** 42.2 (16.7)

Krum H et al.	2012	RCT	Australia	12 Months	**Usual Care** (n = 217) 73 (11) **Usual Care + Intervention** (n = 188) 73 (10)	405	**Usual Care** 54/36 **Usual Care + Intervention** 52/38	II–IV	Criteria LVEF <40% **Usual Care** 34.9 (23.5) **Usual Care + Intervention** 37.2 (14.1)

Lynga P et al.	2012	RCT	Sweden	12 months	73	319	**Control:** 74/26 **Intervention:** 76/24	III–IV	Criteria LVEF <50% 57% had LVEF <30%

Mortara A et al.	2008	RCT	United Kingdom, Poland, Italy	12 months	60 ± 11	461	**Control:** 83/17 **Intervention:** 86/14	II–IV	Criteria LVEF <40% Mean LVEF 29 (7)

Seto E et al.	2012	RCT	Canada	6 months	55.1 (13.7)	100	**Control:** 76/24 **Intervention:** 82/18	II–IV	Criteria LVEF <40%

Soran OZ et al.	2008	RCT	USA (3 distinct medical centres)	6 months	**Alere** 76.9(7.1) **Standard care** 76(6.8)	315	**Alere** 31/69 **SC** 61/39	II–III	Criteria LVEF <40% **Alere** 24.3(8.8) **SC** 23.8(8.7)

Wakefield BJ et al.	2007	RCT	USA	12 months	69.3 (9.6)	148	**Videophone** 88/12 **Telephone** 94/6 **Usual care** 100/0	II–IV	Mean LVEF 41.4% (Range 6–81%)

Woodend AK et al.	2008	RCT	Canada	3 months	68	121	**Telehome** 74/27 **Usual care** 70/30	II–IV	N/A


**Abbreviations:** RCT, randomised controlled trial; NYHA, New York heart association; LVEF, left ventricle ejection fraction; USA, United States of America; UK, United Kingdom; PDA, personal digital assistant; RTM, remote telemedical management; NTM, nurse telemonitoring; HNV, home nurse visit; MG, monitored group; SC, standard care; N/A, not available/not known/not mentioned.

**Table 4:** Description of intervention in randomised clinical trials.


AUTHOR	TYPES OF INTERVENTION

Villani et al.	Telemonitoring using a handheld PDA connected with the monitoring center

Pedone et al.	Telemonitoring system that receives oxygen saturation, heart rate, and blood pressure readings with telephone support

Kenealy et al.	Using a small device to measure and input data daily (include weight, blood pressure, and oxygen level) compared with usual care

Pekmezaris et al.	Tailored telehealth self-monitoring (consists of daily vital signs monitoring and weekly video visit) compared with comprehensive outpatient management

Riegel B et al.	Telephonic case-management and use of software programs to identify important clinical data such as patients’ worsening of symptoms, knowledge and medical needs.

Benatar D et al.	Transtelephonic home monitoring devices to measure vital signs. Nurse evaluates objective data and conducts telephone assessments, titrates medication therapy and educates patients

Dar O et al.	Daily telemonitoring of signs and symptoms (e-weigh scale, automated blood pressure cuff, pulse oximeter, and a control box connected to phone line. Data were reviewed on a daily basis by a HF nurse. Any abnormal results alert the health personnel who would call the patients for further assessment and medical advice

Koehler F et al.	Patients were provided with telehealth patient station in their home. Weekly virtual nursing visits and monitoring of symptoms and vital signs on daily basis.

Jerant AF et al.	Scheduled phone call from study nurse and video-based telecare

Kurtz B et al.	Automated home-based self-monitoring using conventional telephone weekly. The algorithm is able to advise patients medically based on their symptoms. Patients are scheduled for three visits to the clinic in a year.

Melin M et al.	Utilised telemonitoring system. E-weighing scale is connected to OPTILOGG system (tablet computer and custom software), which also contains education module about HF and patients are instructed to input their symptoms every 5 days

Antonicelli R et al.	Telephone support system weekly by HF team to collect information on vital blood signs, urine output and body weight, as well as symptoms and treatment adherence. A weekly ECG was also recorded. Patients and caregivers underwent training courses to apply the home study protocol and correct use of equipment. Therapeutic regimen was regularly assessed and altered as necessary based on telemonitored data or telephone interviews.

Baker DW et al.	Daily weighing to guide diuretic self-adjustment, including an individualized pain developed with the patient clinicians. Symptoms monitoring, intensive education and self-care training through 5–8 follow up phone calls in a month.

Blum K et al.	Remote monitoring of daily weights, blood pressure, heart rate and 15-second heart rhythm strip using Phillips Electronics E-care System. Data was then transmitted wirelessly and compared to individually assigned parameters based on subjects’ admission and evaluations. Any needed adjustments including medication dosage and/or any readings outside of the normal parameter was then done by a nurse practitioner.

DeWalt DA et al.	Structured telephone support through scheduled follow-up phone calls (day 3, 7 and every 7 days, and monthly during months 3–6) preceded by educational session and allotment of educational booklets with clinical pharmacist or health educator regarding signs of HF exacerbation, daily weight assessment and diuretic dose adjustment. Phone calls were done to reinforce these educational points.

Ferrante D et al.	Patients were followed up with a telephone intervention by specialised nurses. Patients were initially called every 14 days and then adjusted according to the severity of each patient. Nurses were allowed to adjust short-term changes in diuretics and to suggest unscheduled visits to the attending cardiologist. Control group continued treatment with their cardiologist in the same manner as the intervention group.

Goldberg LR et al.	Patients in the intervention group received continued standard outpatient heart failure therapy plus AlereNet system or standard outpatient heart failure therapy. The AlereNet system includes an electronic scale and an individualised symptom response system (DayLink monitor) linked via a standard phone line to a computerized database monitored by trained cardiac nurses. Patients were instructed to weigh themselves and respond to yes/no questions about HF symptoms twice daily. The nurses contacted the patient as necessary to verify any changes observed in symptoms or weight.

Mizukawa M et al.	Patients in all groups were provided with a notebook to record daily self-monitoring data such as weight, blood pressure, and pulse. Patients in the usual care group received one standard education session at enrollment using a pre-existing booklet and received HF treatment provided by their physician. Patients in the intervention groups (self-management and collaborative management) received disease management programs for 12 months. In addition, patients in the CM group received telemonitoring intervention, in which a nurse checked data and called patients as needed for 12 months. Each patient received noninvasive physiologic telemonitoring devices to measure BP, pulse rate, and body weight daily. The data were transmitted to the nurse’s computer and checked daily by trained nurses. The nurses also arranged physician visits or contacted the patient care manager for care coordination as needed.

Krum H et al.	Usual care involved standard general practice management of heart failure according to the National Heart Foundation of Australia/Cardiac Society of Australia and New Zealand Heart Failure Management Guidelines. In addition to UC, the UC plus intervention group received ongoing support by touchtone telephone using the TeleWatch system. This telemedicine system was required to be dialled into by the patient on a monthly basis at which time questions were asked with regard to heart failure clinical status by heart failure specialist nurses. In addition, the patients were able at any time to dial to the system and receive advice about management of their heart failure symptoms or be directed to a general practitioner or an emergency department. Patient information resource, regular newsletters, and an individualised patient diary were also provided for the intervention group.

Lynga P et al.	Telemonitoring using an electronic scale (Zenicor Medical Systems AB) installed in the patient’s home. Quote, “…after weighing, a wireless signal was sent from the scale to a modem plugged into the patient’s telephone. The weight was then automatically transmitted via the telephone network to a central internet-based data server system (Zenicor Medical Systems AB). Hence, the weight could be checked from any computer with internet access. The system sounds an alarm if the patient gains > 2 kg from the target weight and if there is an increase of > 2 kg in 3 days.

Mortara A et al.	Home telemonitoring using a cardiorespiratory recorder and modem, digital blood pressure monitor (UA-767, A&D Company, Tokyo, Japan), and electronic weighing scale. Patients were randomized into usual care and three home telemonitoring groups: (i) monthly telephone contact; (ii) strategy 1 plus weekly transmission of vital signs; and (iii) strategy 2 plus monthly 24 h recording of cardiorespiratory activity.

Seto E et al.	Telemonitoring of body weight and blood pressure (UA UC-321PBT weight scale and UA-767PBT blood pressure monitor, A&D Medical, USA) and ECG recordings (SelfCheck ECG PMP4, CardGuard, Israel) were automatically sent wirelessly to a mobile phone (BlackBerry Pearl 8130, Research in Motion, Canada) via Bluetooth before being transmitted to the data center at the hospital. The cardiologist would call the patients once alerted by abnormal data

Soran OZ et al.	Home-based disease management program (Alere DayLink HF Monitoring System, HFMS) detects early signs and symptoms of HF linked to a standard phone line and into a computerized database run by trained nurses. Patients were instructed to weigh themselves and respond to HF symptoms questions daily. Transmitted data were reviewed daily, and patients were contacted to verify changes in observed symptoms and/or weight. Significant changes in symptoms and/or weight were alerted to attending physicians who then adjust therapeutic changes and/or schedule patient visits accordingly.

Wakefield BJ et al.	Telephone or videophone interviews were conducted weekly by a nurse to assess patients’ reported HF-related symptoms, body weight, blood pressure, ankle circumference that were measured by the patients. Additionally, patients underwent behaviour skill training to optimise self-management, self-monitoring and self-efficacy

Wooden AK et al.	Home-monitoring equipment was installed in the patient’s home. Patients were instructed to measure body weight and blood pressure daily and data will be transmitted to a central station/The 12-lead ECG was also recorded periodically. Video conference with a nurse was conducted weekly to review the patient’s progress and self-care education


**Table 5:** Critical appraisal for systematic reviews using AMSTAR 2 checklist.


SYSTEMATIC REVIEWS	AMSTAR 2 ITEMS																QUALITY OF REVIEW

	1	2	3	4	5	6	7	8	9	10	11	12	13	14	15	16	

Inglis et al.	Y	Y	Y	PY	Y	Y	Y	Y	Y	Y	Y	Y	N	Y	Y	Y	Low

Allida et al.	Y	Y	N	PY	Y	Y	Y	Y	Y	Y	Y	Y	N	Y	Y	N	Low


**Abbreviations:** Y, Yes; PY, Partial Yes; N, No.

Most of the RCTs were performed in developed countries, except a study by Ferrante et al. in Argentina [12]. Mean age of participants ranged from 55–78 years old. The proportion of male participants was higher in the majority of studies, except in the study by Pedone et al. and Jerant et al. [13,14]. Most of the studies included patients with NYHA class II-IV, whereas Inglis et al., Allida et al., Pekmezaris et al., Koehler et al., and Soran et al. only included patients with NYHA class II–III [7,11,15,16,17]. Participants in three other studies [18,19,20] were in NYHA class III–IV. Some studies had reported criteria of LVEF, most of which were under 40%. Mean LVEF of the participants ranged from 21.8% to 44%. Other studies did not report mean LVEF.

In regards to intervention, twenty four of the included trials utilized home-based devices to monitor vital signs and body weight [7,12,13,15,16,17,18,19,20,21,22,23,24,25,26,27,28,29,30,31,32,33,34,35]. The received data are transmitted for review by health personnel or by a built-in algorithm. Adjustments of medical therapies are made whenever necessary. Aside from telemonitoring, some studies employed a combined method of interventions, that is, structured telephone calls or video visits [12,15,18,19,20,28,29,30,31,32,33,34,35,36]. Two studies used telemedicine only to substitute face-to face consultation [7,21]. Table 4 summarizes the types of interventions utilized in the trials included in this study. Duration of intervention varied from 1 to 36 months. A sufficient level of bias in the twenty-two RCTs was detected using the PEDro tools. Quality of the other three RCTs was fair. Blinding of participants was not possible in this type of study due to the nature of intervention (see Table 6).

**Table 6:** Critical appraisal for RCTs using PEDro scale.


RCTS	PEDRO SCALE ITEMS											TOTAL SCORE	QUALITY

	ELIGIBILITY	1	2	3	4	5	6	7	8	9	10		

Villani et al.	Yes	1	0	1	0	0	0	1	1	1	0	5/10	Fair

Pedone et al.	Yes	1	1	1	0	1	0	1	0	1	1	7/10	Good

Kenealy et al.	Yes	1	1	1	0	0	0	1	1	1	1	7/10	Good

Pekmezaris et al.	Yes	1	1	1	0	0	0	1	1	1	1	7/10	Good

Riegel B et al.	Yes	1	1	1	0	1	0	1	1	1	1	8/10	Good

Benatar D et al.	Yes	1	0	1	0	0	0	1	1	1	1	6/10	Good

Dar O et al.	Yes	1	0	1	0	0	1	1	1	1	1	7/10	Good

Koehler F et al.	Yes	1	0	1	0	0	1	0	1	1	1	6/10	Good

Jerant AF et al.	Yes	1	1	1	0	0	1	1	1	1	1	8/10	Good

Kurtz B et al.	Yes	0	0	0	0	0	0	1	1	1	1	4/10	Fair

Melin M et al	Yes	1	0	1	0	0	1	0	1	1	1	6/10	Good

Antonicelli R et al	Yes	1	0	1	0	0	0	1	1	1	1	6/10	Good

Baker DW et al	Yes	1	0	1	0	0	1	1	0	1	1	6/10	Good

Blum K et al	Yes	1	1	0	1	0	0	0	1	1	1	6/10	Good

DeWalt DA et al	Yes	1	1	1	1	0	0	0	1	1	1	7/10	Good

Ferrante D et al	Yes	1	0	1	0	0	0	1	1	1	1	6/10	Good

Goldberg LR et al	Yes	1	0	1	0	0	1	1	1	1	1	7/10	Good

Mizukawa M et al	Yes	1	0	1	0	0	0	1	1	1	1	6/10	Good

Krum H et al	Yes	1	0	1	0	0	1	1	1	1	1	7/10	Good

Lynga P et al	Yes	1	0	1	0	0	0	1	1	1	1	6/10	Good

Mortara A et al	Yes	1	1	1	0	0	1	1	0	1	1	7/10	Good

Seto E et al	Yes	1	1	1	0	0	0	1	0	1	1	6/10	Good

Soran OZ et al	Yes	1	1	0	1	0	0	1	1	1	1	7/10	Good

Wakefield BJ et al	Yes	1	0	1	0	0	0	0	1	1	1	5/10	Fair

Wooden AK et al	Yes	1	0	1	0	0	0	1	1	1	1	6/10	Good


### Study Results

Studies incorporating either telemonitoring devices or telephone-based monitoring systems were compared with standard outpatient visits (usual care). The results of the included studies are presented in Tables 7 and 8. Mortality rate was evaluated in eighteen studies. Four out of these eighteen studies demonstrated beneficial results in mortality rate after participants received telemedicine intervention for a period ranging from 6 to 12 months. Inglis et al. found that both structured telephone support (STS) (RR 0.87; 95% CI 0.77–0.98) and non-invasive home telemonitoring (HT) (RR 0.80; 95% CI 0.69–0.94) significantly reduced mortality compared with usual care during the six-month study period [7]. Similar result was shown by Pedone et al. and Goldberg et al. [RR 0.51 (95% CI 0.26–0.98), RR 0.43 (95% CI 0.22–0.84); p = 0.0142] after a six-month duration of follow-up [13,20]. Longer duration of follow-up up to a year also proved to be beneficial in reducing mortality [26]. Other studies showed that the use of telemedicine had no significant effect on mortality rate [12,15,18,19,30,31,32,33,34,35,36].

**Table 7:** Results of the systematic review included in the study.


STUDIES	STUDY RESULTS			

	MORTALITY	HR-QOL	ALL-CAUSE HOSPITALISATION	HF-RELATED HOSPITALISATION

**Inglis et al.**	**STS** RR 0.87 (95% CI 0.77–0.98); I^2^ = 0% **HT** RR 0.80 (95% CI 0.69–0.94); I^2^ = 24%	N/A	**STS** RR 0.95 (95% CI 0.90–1.00); I^2^ = 47% **HT** RR 0.95 (95% CI 0.89–1.01); I^2^ = 71%	**STS** RR 0.85 (95%CI 0.77–0.93); I^2^ = 27% **HT** RR 0.71 (95%CI 0.60–0.83); I^2^ = 20%

**Allida et al.**	N/A	MLHFQ MD -0.10 lower in the intervention group (95% CI -2.35 to 2.15); I^2^ = 61%)	N/A	OR 0.74 (95% CI 0.52–1.06); I^2^ = 0%


**Abbreviations:** HR-QoL, health-related quality of life; STS, structured telephone support; HT, non-invasive home telemonitoring; MLHFQ, Minnesota Living with Heart Failure Questionnaire; RR, relative risk; HR, hazard ratio; CI, confidence interval; OR, odd ratio; N/A, not available.

**Table 8:** Results of the randomised control trials included in the study.


STUDIES	STUDY RESULTS			

	MORTALITY	HR-QOL	ALL-CAUSE HOSPITALIZATION	HF-RELATED HOSPITALIZATION

**Villani et al.**	RR 0.56 (95%CI 0.20–1.51), 5/40 vs 9/40,p > 0.05 at 1-year follow-up	N/A	N/A	RR 0.52 (95%CI 0.30–0.89) 12/40 vs 23/40, p < 0.03, at one-year of follow up

**Pedone et al.**	RR 0.51 (95% CI 0.26–0.98) at 6-month follow-up	N/A	RR 0.30 (95% CI 0.12–0.67) at 6-month follow-up	RR 0.48 (95% CI 0.14–1.45) at 6-month follow-up

**Kenealy et al.**	RR 0.63 (95%CI 0.21–1.82) at 6-month follow-up	Coefficient of interaction (telecare vs usual care) of 0.47 (p = 0.63) after 6 months of follow up using the SF-36 (Mental component score)	95 vs 63 (p-value unavailable) at 6-month follow-up	N/A

**Pekmezaris et al.**	N/A	TSM: 62.7 at baseline and 36.3 after 90 days vs COM: 59.9 at baseline and 27.8 after 90 days, p = 0.50 using MLHFQ	Binary analysis: RR 0.92 (95% CI 0.57–1.48, p = 0.73) during 90 days of follow up; Non-binary analysis mean (62): 0.78 (1.3) TSM vs 0.55 (0.9) COM, p = 0.03	Binary analysis: RR 1.27 (95% CI 0.44–3.6, p = 0.65) during 90 days of follow up; Non-binary analysis mean (62): 0.15 (0.47) TSM vs 0.16 (0.41) COM, p = 0.76

**Riegel B et al.**	N/A	N/A	**Telemedicine** 0.45(0.73) vs **usual care** 0.61(0.88) at 3-month follow-up, p = 0.09; **Telemedicin**e 0.62(0.88) vs **usual care** 0.87(1.1) at 6-month follow-up, p = 0.03	**Telemedicine** 0.17 (0.43) vs **usual care** 0.31(0.64) at 3-month follow-up, p = 0.03; **Telemedicine** 0.21(0.5) vs **usual care** 0.4(0.77) at 6-month follow-up, p = 0.01

**Benatar D et al.**	N/A	MLHFQ Pre vs post intervention HT group: 77.92 (10.30) vs 51.64(17.36), p < 0.01 Pre vs post intervention home nurse visit: 77.1(8.52) vs 57.72 (16.24), p < 0.01 Between-group p = 0.98	13 vs 24, p ⩽ 0.001 at 3-month follow-up 38 vs 63, p ⩽ 0.05 at 6-month follow-up 75 vs 103, p = 0.12 at one-year follow-up	N/A

**Dar O et al.**	N/A	N/A	36 % vs 81% (p = 0.01) at 6-month follow-up	N/A

**Koehler F et al.**	HR 0.97 (95% CI 0.67–1.41), p = 0.87	SF-36 (physical functioning) mean score (46) 54.3 (1.2) vs 49.9 (1.2), p = 0.01 after 12 months	N/A	N/A

**Jerant AF et al.**	N/A	N/A	RR 0.36 (95% CI 0.21–0.62)	N/A

**Kurtz B et al.**	Risk reduction 22% vs 44%, p = 0.04 at one year follow-up	N/A	N/A	

**Blum K et al.**	RR 1.07 (95%CI 0.79–1.44) at 4-years follow-up	MLHFQ Scores improved over the years within UC and MG (p < 0.001), but no difference between UC & MG	RR 1.06 (95%CI 0.90–1.24)	Mean HF hospitalizations per subject MG 2 ± 2 vs. UC 3 ± 3 (p = 0.76)

**DeWalt DA et al.**	RR 0.79 (95% CI 0.18–3.37)	MLHFQ Difference between scores in IG and CG 3.5 points (95% CI -4–11), p = 0.36	Crude all-cause hospital admission or death IRR 0.69 (95% CI 0.40–1.19)	Unadjusted IRR 0.79 (95% CI 0.42–1.5)

**Ferrante D et al.**	**Intervention vs. Control** At 1 year: RR 0.94 (0.77–1.16); p = 0.586 At 3 years: RR 1.02 (0.87–1.2); p = 0.73	**Intervention vs. Control** MLHFQ Global score: 30.6 vs. 35, p = 0.001 Physical domain: 11.2 vs. 12.8, p –0.007 Emotional domain: 6.7 vs. 7.9, p = 0.002	N/A	**Intervention vs Control** At 1 year: 174 (22.9%) vs 220 (29%); RR 0.73 (0.6–0.9); p = 0.002 At 3 years: 217 (28.9%) vs 266 (35.1%); RR 0.72 (0.6–0.87); p = 0.0004

**Goldberg LR et al.**	**Intervention vs Control** 11 (8%) vs. 26 (18.4%), number needed to treat 9.7, p < 0.003 RR: 0.43 (95%CI 0.22–0.84); p = 0.0142	**Intervention vs Control** MLHFQ (mean ± SD) –27.8 ± 23.8 vs -23.3 ± 26.9, p = 0.22	**Intervention vs Control** (mean ± SD) average utilisation per patient per month 0.19 ± 0.46 vs 0.2 ± 0.3, p = 0.28	**Intervention vs Control** (mean ± SD) average utilisation per patient per month 0.08 ± 0.24 vs. 0.11 ± 0.26, p = 0.28

**Mizukawa M et al.**	15% vs. 15.8% RR: 1.1 (95%CI 0.25–4.83), p = 0.8996	Using MLHFQ The CM group had better improvement with statistical significance vs UC group at 18 months (p = 0.014) and at 24 months (p = 0.016) vs SM group at 18 months (P = 0.044); vs baseline at 6 months (p = 0.002), 12 months (p = 0.012), 18 months (p = 0.003) and 24 months (p = 0.018)	60% vs. 68.4% RR: 0.87 (95%CI 0.54–1.40); p = 0.5843	20% vs. 57.9% HR: 0.29 (95% CI, 0.09–0.92; p = 0.035)

**Krum H et al.**	**Usual Care** 16/209 (7.6%) **Usual Care + Intervention** 17/170 (10%) Unadjusted HR: 1.3 (95%CI 0.65–2.77, p = 0.43) Adjusted HR: 1.36 (95% CI 0.63–2.93, p = 0.439)	N/A	**Usual Care** 114/204 (55.8%) **Usual Care + Intervention** 74/161 (45.9%) Unadjusted HR: 0.71 (95%CI 0.53–0.95, p = 0.021) Adjusted HR: 0.67 (95%CI 0.50–0.89, p = 0.006)	**Usual Care** 35/204 (17.2%) **Usual Care + Intervention** 23/161 (14.3%) Unadjusted HR: 0.81 (95%CI 0.44–1.38, p = 0.43) Adjusted HR: 0.78 (95%CI 0.45–1.33, p = 0.36)

**Lynga P et al.**	8/153 vs. 5/166, HR 0.57 [0.19–1.73], p = 0.32	N/A	84/153 vs. 79/166, HR 0.83 [0.61–1.13], p = 0.24	70/153 vs. 70/166, HR 0.90 [0.65–1.26], p = 0.54

**Mortara A et al.**	7/94 vs. 9/160, RR 1.32 (95% CI 0.51–3.44)	N/A	34/94 vs 48/160, RR 1.21 (95% CI 0.84–1.72)	17/94 vs 28/160, RR 1.03 (95% CI 0.60–1.78)

**Seto E et al.**	3/50 vs 0/50, RR 7.00 (95% 0.37–132.10)	MLHFQ Control Group: p = 0.9; Intervention Group: p = 0.02; Between group post study: p = 0.2; Between group change scores data: p = 0.05	14/50 vs 10/50, RR 1.40 (95% CI 0.69–2.85)	N/A

**Soran OZ et al.**	RR 0.63 (95% CI 0.30–1.29)	N/A	RR 1.10 (95% CI 0.86–1.41)	**Unadjusted HR** 0.78 (95% CI 0.48–1.27); **Adjusted HR** (NYHA, B-blocker use, Sex, Na levels) 0.71 (95% CI 0.43–1.17)

**Wakefield BJ et al.**	HR 1.04 (95% CI 0.49–2.24; p = 0.91) at 12-month follow up	MLHFQ p = 0.0002 (changes over 6 months within all groups). Between-group p value not significant	OR 0.49 (95% CI 0.24–0.98; p = 0.04) at 12-month follow up	OR 0.58 (95% CI 0.21–1.56; p = 0.28) at 12-month follow up

**Woodend AK et al.**	RR 1.19 (95% CI 0.34–4.22)		RR 1.06 (95% CI 0.97–1.16)	


**Abbreviations:** HR-QoL, health-related quality of life; MLHFQ, Minnesota Living with Heart Failure Questionnaire; SF, Short Form; TSM, telehealth self-monitoring; COM, comprehensive outpatient management; MD, mean difference; RR, relative risk; HR, hazard ratio; CI, confidence interval; OR, odd ratio; N/A, not available.

Five studies reported significant improvements in HR-QoL before and after intervention of telemedicine, although none were statistically significant when compared between groups. Allida et al. [13], Pekmezaris et al. [15] and Benatar et al. [19] used the Minnesota Living with Heart Failure Questionnaire (MLHFQ), while Kenealy et al. [17] and Koehler et al. [14] used the Short Form-36 Survey (SF-36) to evaluate QoL. An interesting result was shown by Pekmezaris et al., in which the HR-QoL of the comprehensive outpatient management group showed greater improvement from baseline compared to the telemedicine group [16].

The majority of studies reported lower hospitalisation rates in groups receiving telemedicine. Inglis et al. showed that both STS and HT had similar insignificant effects on all-cause hospitalisation (RR 0.95; 95% CI 0.90–1.00) and (RR 0.95; 95% CI 0.89–1.01), respectively [8]. A similar result was shown by Koehler et al. (HR 1.12; 95% CI 0.91–1.37) [14]. Contradictory results were demonstrated by Pedone et al., Riegel B et al., Benatar et al. and Dar et al. [13,23,24,25]. These studies reported that all-cause hospitalisation was reduced significantly after six months of follow-up. Kenealy et al. also found a higher proportion of all-cause hospitalisation in patients receiving telecare intervention compared to usual care group [22].

Meanwhile, Inglis et al. had also observed significant reduction in the HF-related hospitalisation in patients receiving STS and HT (RR 0.85; 95%CI 0.77–0.93 and RR 0.71; 95%CI 0.60–0.83, respectively) [8]. These results are in line with studies by Villani et al., Riegel et al. and Kurtz et al. [21,23,26]. A longer duration of study up to three years also resulted in significant benefit in reducing HF-related hospitalisation as demonstrated by Ferrante et al. [12]. However, insignificant improvements on the HF-related hospitalisation were shown by Allida et al. [13] (OR 0.74; 95%CI 0.52–1.06) and Pedone et al. [13] (RR 0.48; 95% CI 0.14–1.45). Pekmezaris et al. did binary and non-binary analysis on both all-cause and HF-related hospitalisation [16]. There were no significant reductions in all-cause hospitalisation (RR 0.92; 95% CI 0.57–1.48, p = 0.73) and HF-related hospitalisation (RR 1.27; 95% CI 0.44–3.6, p = 0.65).

## Discussion

The burden on health care systems worldwide has increased enormously during the COVID-19 pandemic. Patients have become more reluctant to seek medical help for their illnesses due to fear of infection [8]. Consequently, for decompensated HF patients, reluctance to seek medical help may lead to late medical or non-medical treatments that are to no avail. This necessitates the implementation of technology in clinical settings to safely and effectively deliver health care in a timely manner. Telemedicine has been increasingly utilized during the COVID-19 pandemic with high levels of satisfaction from patients and healthcare providers [37,38]. The European Society of Cardiology has recognized the potential of telemedicine and encouraged its use whenever preferable, while containing the spread of infections [39].

We investigated the outcomes of telemedicine on heart failure patients with NYHA class II to IV in our study. Previous study had found that rates of all-cause mortality and all-cause hospitalisations were higher in patients with NYHA class II–IV [40]. Additionally, we included patients aged 45 years old and above. As discussed earlier, the demography of heart failure patients has gradually transitioned towards the older populations. This is evident in our included studies where the mean age of HF patients ranged from 45 to 78 years old. Older adults are increasingly using technology, although they still lag behind compared to those aged between 18 and 64. They face unique issues related to physical and cognitive functioning that hinders them from finding and using appropriate health information and technology [41]. Therefore, it is important to bear in mind that technology-based health care delivery services should be user-friendly and meet demands of users across diverse ages.

**Figure 2:**
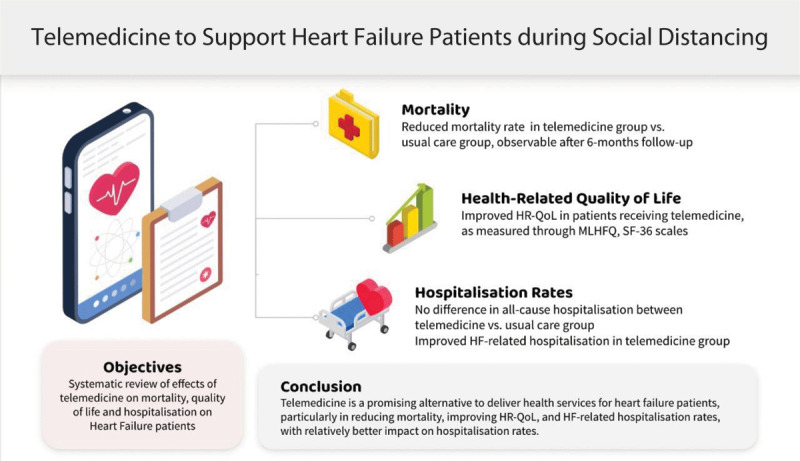
Central Illustration highlighting summary of this systematic review findings. HF: Heart Failure, HR-QoL: Health-Related Quality of Life; MLHFQ: Minnesota Living with Heart Failure Questionnaire.

Methods of delivery of telemedicine vary amongst the included studies. Out of 27 studies, 92% had utilised telemonitoring system that receive vital signs readings such as blood pressure, oxygen saturation level, and weight [7,12,13,15,16,18,19,20,21,22,23,24,25,26,27,28,29,30,31,32,33,34,35,42]. Important clinical data such as patients’ worsening of symptoms and medical needs are identified. According to the World Health Organization, telemedicine, by definition, means ‘the provision of healthcare services at a distance with communication conducted between healthcare providers seeking clinical guidance and support from other healthcare providers (provider-to-provider telemedicine); or conducted between remote healthcare users seeking health services and healthcare providers (client-to-provider telemedicine)’ [43]. In this review, the use of this telemonitoring is often coupled with structured telephone support or video visits from healthcare personnel on a regular basis to check on the patients as well as to give medical services accordingly based on these data whenever deemed necessary. One study [14] had performed scheduled group telephone call or video-based telecare and another study [11] utilised the internet and web-based programmes on smartphone and mobile devices to deliver education. The ESC e-Cardiology Working Group position paper emphasised that patient-education programmes should be part of solutions to challenges in digital health implementation in Europe [44]. As highlighted in a review by Inglis et al., home telemonitoring yields lower risk of mortality and HF-related hospitalisation compared to structured telephone support. In this study, it was demonstrated that telemedicine delivers a relatively better impact on HF-related hospitalisation than mortality. Figure 2 illustrates the central result of this study.

### Mortality Rate

Heart failure is a life-limiting illness for many patients, with half of patients with severe HF dying within one year of diagnosis [45]. One study found that 57% of mortality rates in HF patients were categorised as cardiovascular, with coronary heart disease as the main cause of death in 63% of cardiovascular deaths [46]. Furthermore, populations with HF are shown to be more vulnerable to infections, which has emerged as a key immediate cause of death [47]. Another study showed that pre-existing HF is a risk factor for more severe clinical courses of COVID-19 and an independent predictor of in-hospital mortality [48]. Coincidentally, patients with HF are at a higher risk of contracting COVID-19 as they require continuous care in standard outpatient or inpatient settings. In a traditional setting, patients have to physically leave their homes and present themselves to the hospital [49]. Studies has shown that patient-to-patient COVID-19 transmissions in hospitals might reach 52%, with 40–50% of those cases resulting in further transmissions [50]. Furthermore, prior to the pandemic, evidence has shown that HF patients are also at increased risk of hospital-acquired pneumonia (HAP). A study by Tada et al. found that the prevalence of HAP in hospitalised acute heart failure patients was as high as 8% and they developed a higher rate of all-cause mortality, as well as worsening of symptoms [51]. Telemedicine can mitigate such risk by providing healthcare services for HF patients from their own comfort.

Our study found a reduction in the mortality rate of patients in the telemedicine group compared to the usual care group, although only some showed statistically significant results. Favourable outcomes were observable after six months of follow-up period. A systematic review by Inglis et al. analysed in our study involved the largest number of study participants and concluded positive results on all-cause mortality [7]. However, we rated low level of confidence in risk of bias analysis as the majority of the involved studies failed to elucidate methods of random sequence generation in their RCTs and concealment of allocations. This is somewhat similar to our analysis of risk of bias. The nature of this intervention prevents blinding of study participants. Blinding of outcome assessors and therapists can be made possible. Therefore, further studies of RCTs producing exceptional qualities are warranted. The Telemedical Interventional Monitoring in Heart Failure (TIM-HF) trial conducted by Koehler et al. [42] did not observe a lower rate of mortality in HF patients with NYHA class II–III but the author agreed that it would be potentially beneficial for certain heart failure populations.

Nevertheless, the positive outcome is supported by Armaignac et al. who showed that telemedicine intervention decreased overall mortality rate by 20% in the progressive care unit without substantial cost incurrences [52]. In 2010, six systematic reviews, reviewed by Ekeland et al. also reported either a reduction in mortality rates or equivalent rates between telehealth and usual care groups [53]. It is therefore safe to say that the safety of telemedicine is comparable to that of usual care in heart failure patients. Effective interventions include monitoring of vital signs at home with telephone follow-up by nurses for case adjustments and managements. On the contrary, telehealth patients were more likely to experience emergency admission or death by 1.34-fold in the study by Steventon et al. This study, however, included a wider range of populations such as those with chronic obstructive pulmonary disease (COPD), heart failure, and diabetes [54].

### HR-QoL

Besides having physical limitations (e.g., shortness of breath, pain, fatigue, and restrictions regarding daily living activities), patients with advanced cardiac failure also suffer from psychosocial limitations (e.g., fear and social isolation), which contribute to the decrease of QoL. [47]. Another study mentioned that individuals with heart failure have markedly impaired QoL compared to other chronic diseases, as well as compared to the healthy population, which is related to high hospitalisation and mortality rates [55]. The NYHA functional class was found to be the most dominant predictor of reduced quality of life amongst heart failure patients [56]. Therefore, it is important to improve QoL in heart failure patients and determine its impact on patients’ daily lives.

Literatures included in this study showed improvement on the HR-QoL of HF patients who received delivery of services via telemedicine [17,18,33]. Similar results were shown in populations with lung cancer who also received telemedicine interventions, especially in patients with long-term follow-up [57]. Meta-analysis by Li et al. also showed better quality of life in cancer survivors, with most improvement seen in the breast cancer group using application-based intervention [57]. However, there are also some studies that show telemedicine is not superior to usual care. Interestingly, Pekmezaris et al. found that HF patients had a non-significant improvement of QoL after receiving comprehensive outpatient management compared to those who had telehealth interventions [16]. Discomfort of using telemonitoring devices may arise as patients are being frequently reminded of their chronic conditions or having to take the measurements themselves. A total of 759 patients with COPD, diabetes, or heart failure were analysed by Cartwright et al. after receiving 12 months of telemedicine. No significant differences in regards to HR-QoL, anxiety, and depressive symptoms were demonstrated [58]. Ultimately, this also means that technology-based intervention does not negatively impact patients’ quality of life.

### Hospitalisation Rate

Data from the USA showed that in 2005–2018, heart failure was only second to sepsis as the most common cause of hospitalisation [59]. A study with 8,603 HF patients showed that recurrent HF hospitalisations increased risk of cardiovascular mortality by 2.65-fold, which was positively correlated with the number of visits [60]. Pneumonia or other respiratory problems, cardiac ischemia or arrhythmia, and worsening of renal function were the most common precipitating factors. These were found in more than 60% of a cohort of HF patients and independently associated with higher in-hospital and follow-up mortality [61].

Earlier studies investigating telemedicine and hospitalisation rate had shown positive outcomes. A study performed by O’Connor et al. showed promising results after three years of telehealth which lowered hospital readmission rate of HF patients by approximately 14%. Based on these results, the University of Pennsylvania Health System incorporated telehealth in the readmission reduction program [62]. The results of our studies on all-cause and HF-related hospitalisations supported previous evidence. Decreased patterns were seen in most studies, albeit not statistically significant. With increased supervision available via telemonitoring, health care teams were likely to check on the patients more frequently than they otherwise would have [22]. This may have led to early detection of abnormal findings and timely intervention by site coordinators based on the reported symptoms and weight. Non-significant improvement in the rate of rehospitalisation might be due to the lack of interactive communication [63] or poor adherence [64]. Although, Kotooka and colleagues had found a high adherence rate at about 90% after 12 months during the 15 month period of study, they included low-risk HF patients. DeBusk et al. also demonstrated no reduction in rehospitalisation rates in low-risk HF patients compared with usual care. However, the study was single-centred and should be interpreted with care [65]. On the other hand, Mizukawa et al. integrated collaborative self-management through interactive communication via a telemonitoring system and observed a significant improvement in QoL and readmission rate. Therefore, home telemonitoring care systems should not only function as surveillance, but also patient-centered and comprehensive to improve its effectiveness and partnership between patients and healthcare professionals.

Additionally, follow-up visits performed in-person and telemedicine was shown to cause similar reduction of hospitalisation rates compared with no follow-up visits during the COVID-19 pandemic [66]. However, several studies have observed reduction of visits during the COVID-19 pandemic (ranging from 30–66% in different countries), which leads to a subsequent increase in HF mortality [48]. Therefore, extra attention should be directed during this pandemic period as fear against COVID-19 might negatively affect patients’ compliance to scheduled appointments and medication [67].

### Telemedicine in Low-to-Middle Income Countries

Newly existing technologies have made it possible for health delivery services to be relatively more accessible and quick for both individuals and healthcare personnel. The ultimate goal is to achieve an equitable health coverage throughout the Nation in the most cost-effective way possible. However, this does not come without big challenges, one of which is uneven distribution of technology and internet connection. According to the data provided by The Indonesian Ministry of Information and Communication, there are more than 150 million internet users (56% of total population) in Indonesia in 2019 [68]. Most of the internet users and development are located in the Java Island (95.3 million users). Those without access to internet are located in remote areas with no available infrastructure. In Papua and Sulawesi Island, the eastern part of Indonesia, only about 18.6 million people uses the internet [69]. Since 2015, the Indonesian Ministry of Communication has implemented Universal Service Obligation to provide universal coverage of telecommunication across all parts of Indonesia. The programs include development of infrastructures and provision of internet package data in the rural areas. Additionally, in 2017, the Indonesian Ministry of Health has launched the first national telemedicine service called “*TEMENIN*” (*Telemedicine Indonesia*) that serves to provide four basic functions including tele-radiology, tele-electrocardiography, tele-ultrasonography, and teleconsultation. This has been integrated with the referral system and the Indonesian National Health Service. Before the COVID-19 pandemic, the telemedicine was supported by almost 140 health centres (including primary care providers and hospitals) with almost 4 million users in total. The name changed to “*KOMEN*” (*Konsultasi Medis Online)* during the pandemic, although the function remains the same. In 2020, its users rose up to 15 million to monitor self-isolated COVID-19 patients. This number is expected to rise with extra support from the government, advancement of technology, and increased availability of internet connection [70]. Furthermore, Blueprint for Digital Health Transformation Strategy has also been developed by Ministry of Health in 2021 as the potential of digitization of health care is increasingly recognized [71]. A digital innovation called HARKIT I-Care has also been developed by the National Cardiovascular Center Harapan Kita. This smartphone-based application is used to monitor patients with cardiovascular diseases as a secondary preventive strategy and clinical trial is still underway [72,73].

Although telemedicine can be a promising intervention, its implementation might be complicated by several factors. Apart from internet connectivity and coverage, digital literacy is very limited especially among the elderly, low- to middle-income population. It is known that the prevalence of heart failure increases above the age of 60 years old [74]. During the implementation of HARKIT I-Care, patients and caretakers go through several trainings to enable them to fully reap the benefits of telemedicine application. In terms of cost-effectiveness, telemedicine-based intervention in other chronic conditions is related to significantly lower costs compared to standard outpatient treatment. A scoping review for telemedicine implementation in Asian countries (including India, China, Singapore, Japan, and Thailand) shows reduced overall costs of treatment with enhanced effectiveness of health services by saving time and travel costs [75]. The telemedicine-based diabetic retinopathy in Singapore was shown to have saved approximately S$173 per person while generating similar quality-adjusted life-years [76]. Research on the cost-effectiveness of telemedicine specifically for heart failure in developing countries is still scarce as the implementation of telemedicine for heart failure is still low. However, studies in developed countries have shown increased cost-effectiveness up to 35% [77,78].

### Take-Home Messages

The future of heart failure management has never been more promising with the current advances in telemedicine and its benefits as shown from existing evidence, including ours. However, to ensure a sustainable, cost-effective, and most importantly patient-centred care for heart failure patients, key stakeholders including policy makers, community leaders, healthcare professionals, academia, technology and business developers and patients themselves, need to be thoroughly invested in the implementation of telemedicine. The future of telemedicine for heart failure, especially in developing countries are grossly expandable – not only telemonitoring and teleconsultation, but also potentially telerehabilitation, use of teleconferencing to involve multidisciplinary carers and specialistic advice, to potentially the implementation of AI-assisted medical care [79,80,81]. Indeed, telemedicine could not fully replace traditional, in-person care, however with an accessible, and cost-effective telemedicine means, heart failure patients and related healthcare professionals from developing countries can both deliver and receive optimized remote care with emphasis on efficiency, to reduce the burden of heart failure globally.

Result of this study will hopefully encourage people from several important fields such as policy makers, governmental organisation, community leaders, health professionals, academic institutions and educators, technology and business developers, to realise the potentials of telemedicine in heart failure patients.

### Strength and Limitation

To the best of our knowledge, this study yields the most recent updates on the clinical effectiveness of telemedicine in heart failure patients, where the use of telemedicine is rapidly increasing across the globe.

This study has several limitations, due to which interpretation of the results should be done carefully. All of the studies included in this systematic review were performed before the COVID-19 pandemic. The crises had only hit in recent years and hence, only few studies have investigated the outcome of telemedicine in HF patients with significant duration, adequate number of participants and appropriate methodology to extrapolate a high level of clinical evidence during the pandemic. Telemedicine could be a promising solution to provide optimal management of HF patients during this pandemic. As mentioned in the result section, additional 15 studies were identified manually from bibliographies of relevant papers. Since this constitutes more than 50% of the total amount of selected studies, keywords can be improved for future references to allow specific searching of studies that will be better suited to the eligibility criteria. Furthermore, different studies used different techniques and lengths of intervention, as well as varying clinical profiles of the patients studied. Large heterogeneity might exist due to these differences in study designs and population demographics. Implementing telemedicine in developing countries is also still a challenge, which may greatly affect the results. Studies done in developing countries are still limited, as most available studies were done in developed countries where participants have relatively adequate levels of knowledge, discipline, and supporting technology for both patients and healthcare providers.

## Conclusion

The use of telemedicine for heart failure patients has been shown to reduce mortality rate, improve health-related quality of life, and lower hospitalisation rates. Nevertheless, inconsistent results were also observed in these studies, hence careful interpretation is required. Employing telemedicine for heart failure patients who require intensive monitoring will support the need for social distancing during the era of COVID-19 pandemic, as well as protecting patients from the risk of hospital-acquired infections. This interesting field of research will confer numerous benefits in the future, hence warranting further high-quality studies to be performed. Current absence of a standardized guideline of using telemedicine is an opportunity to construct one with a sustainable, cost-effective, and patient-centered plan in mind.

## References

[1] Cohn JN. Plasma norepinephrine and mortality. Clin Cardiol. 1995; 18(3 Suppl I): I9–12. DOI: 10.1002/clc.4960181304 7743697

[2] Hartupee J, Mann DL. Neurohormonal activation in heart failure with reduced ejection fraction. Nat Rev Cardiol. 2017; 14(1): 30–8. DOI: 10.1038/nrcardio.2016.163 27708278 PMC5286912

[3] Lippi G, Sanchis-Gomar F. Global epidemiology and future trends of heart failure. AME Medical Journal. 2020; 5. DOI: 10.21037/amj.2020.03.03

[4] Gaziano TA. Cardiovascular disease in the developing world and its cost-effective management. Circulation. 2005; 112(23): 3547–53. DOI: 10.1161/CIRCULATIONAHA.105.591792 16330695

[5] Bromage DI, Cannatà A, Rind IA, Gregorio C, Piper S, Shah AM, et al. The impact of COVID-19 on heart failure hospitalization and management: report from a Heart Failure Unit in London during the peak of the pandemic. Eur J Heart Fail. 2020; 22(6): 978–84. DOI: 10.1002/ejhf.1925 32478951 PMC7300902

[6] Ponikowski P, Voors AA, Anker SD, Bueno H, Cleland JGF, Coats AJS, et al. 2016 ESC Guidelines for the diagnosis and treatment of acute and chronic heart failure: The Task Force for the diagnosis and treatment of acute and chronic heart failure of the European Society of Cardiology (ESC)Developed with the special contribution of the Heart Failure Association (HFA) of the ESC. Eur Heart J. 2016; 37(27): 2129–200. DOI: 10.1093/eurheartj/ehw128 27206819

[7] Inglis SC, Clark RA, Dierckx R, Prieto-Merino D, Cleland JGF. Structured telephone support or non-invasive telemonitoring for patients with heart failure. Cochrane Database of Systematic Reviews. 2015(10). DOI: 10.1002/14651858.CD007228.pub3 PMC8482064 26517969

[8] Czeisler MÉ, Kristy Marynak M, Kristie EN, Clarke M, Zainab Salah M, Iju Shakya M, et al. Delay or avoidance of medical care because of COVID-19–related concerns. MMWR Morb Mortal Wkly Rep. 2020; 69: 1250–7. DOI: 10.15585/mmwr.mm6936a4 32915166 PMC7499838

[9] Shea BJ, Reeves BC, Wells G, Thuku M, Hamel C, Moran J, et al. AMSTAR 2: A critical appraisal tool for systematic reviews that include randomised or non-randomised studies of healthcare interventions, or both. BMJ. 2017; 358: j4008. DOI: 10.1136/bmj.j4008 28935701 PMC5833365

[10] Cashin AG, McAuley JH. Clinimetrics: Physiotherapy Evidence Database (PEDro) Scale. Journal of Physiotherapy. 2020; 66(1): 59. DOI: 10.1016/j.jphys.2019.08.005 31521549

[11] Allida S, Du H, Xu X, Prichard R, Chang S, Hickman LD, et al. mHealth education interventions in heart failure. Cochrane Database Syst Rev. 2020; 7(7): CD011845–CD. DOI: 10.1002/14651858.CD011845.pub2 32613635 PMC7390434

[12] Ferrante D, Varini S, Macchia A, Soifer S, Badra R, Nul D, et al. Long-term results after a telephone intervention in chronic heart failure: DIAL (Randomized Trial of Phone Intervention in Chronic Heart Failure) follow-up. Journal of the American College of Cardiology. 2010; 56(5): 372–8. DOI: 10.1016/j.jacc.2010.03.049 20650358

[13] Pedone C, Rossi FF, Cecere A, Costanzo L, Antonelli Incalzi R. Efficacy of a physician-led multiparametric telemonitoring system in very old adults with heart failure. J Am Geriatr Soc. 2015; 63(6): 1175–80. DOI: 10.1111/jgs.13432 26031737

[14] Jerant AF, Azari R, Nesbitt TS. Reducing the cost of frequent hospital admissions for congestive heart failure: a randomized trial of a home telecare intervention. Med Care. 2001; 39(11): 1234–45. DOI: 10.1097/00005650-200111000-00010 11606877

[15] Soran OZ, Piña IL, Lamas GA, Kelsey SF, Selzer F, Pilotte J, et al. A randomized clinical trial of the clinical effects of enhanced heart failure monitoring using a computer-based telephonic monitoring system in older minorities and women. J Card Fail. 2008; 14(9): 711–7. DOI: 10.1016/j.cardfail.2008.06.448 18995174

[16] Pekmezaris R, Nouryan CN, Schwartz R, Castillo S, Makaryus AN, Ahern D, et al. A randomized controlled trial comparing telehealth self-management to standard outpatient management in underserved Black and Hispanic patients living with heart failure. Telemed J E Health. 2019; 25(10): 917–25. DOI: 10.1089/tmj.2018.0219 30418101 PMC6784489

[17] Koehler F, Winkler S, Schieber M, Sechtem U, Stangl K, Böhm M, et al. Impact of remote telemedical management on mortality and hospitalizations in ambulatory patients with chronic heart failure: The telemedical interventional monitoring in heart failure study. Circulation. 2011; 123(17): 1873–80. DOI: 10.1161/CIRCULATIONAHA.111.018473 21444883

[18] Mizukawa M, Moriyama M, Yamamoto H, Rahman MM, Naka M, Kitagawa T, et al. Nurse-led collaborative management using telemonitoring improves quality of life and prevention of rehospitalization in patients with heart failure. Int Heart J. 2019; 60(6): 1293–302. DOI: 10.1536/ihj.19-313 31735786

[19] Lyngå P, Persson H, Hägg-Martinell A, Hägglund E, Hagerman I, Langius-Eklöf A, et al. Weight monitoring in patients with severe heart failure (WISH). A randomized controlled trial. Eur J Heart Fail. 2012; 14(4): 438–44. DOI: 10.1093/eurjhf/hfs023 22371525

[20] Goldberg LR, Piette JD, Walsh MN, Frank TA, Jaski BE, Smith AL, et al. Randomized trial of a daily electronic home monitoring system in patients with advanced heart failure: The Weight Monitoring in Heart Failure (WHARF) trial. Am Heart J. 2003; 146(4): 705–12. DOI: 10.1016/S0002-8703(03)00393-4 14564327

[21] Villani A, Malfatto G, Compare A, Della Rosa F, Bellardita L, Branzi G, et al. Clinical and psychological telemonitoring and telecare of high risk heart failure patients. J Telemed Telecare. 2014; 20(8): 468–75. DOI: 10.1177/1357633X14555644 25339632

[22] Kenealy TW, Parsons MJ, Rouse AP, Doughty RN, Sheridan NF, Hindmarsh JK, et al. Telecare for diabetes, CHF or COPD: effect on quality of life, hospital use and costs. A randomised controlled trial and qualitative evaluation. PLoS One. 2015; 10(3): e0116188. DOI: 10.1371/journal.pone.0116188 25768023 PMC4358961

[23] Riegel B, Carlson B, Kopp Z, LePetri B, Glaser D, Unger A. Effect of a standardized nurse case-management telephone intervention on resource use in patients with chronic heart failure. Arch Intern Med. 2002; 162(6): 705–12. DOI: 10.1001/archinte.162.6.705 11911726

[24] Benatar D, Bondmass M, Ghitelman J, Avitall B. Outcomes of chronic heart failure. Arch Intern Med. 2003; 163(3): 347–52. DOI: 10.1001/archinte.163.3.347 12578516

[25] Dar O, Riley J, Chapman C, Dubrey SW, Morris S, Rosen SD, et al. A randomized trial of home telemonitoring in a typical elderly heart failure population in North West London: results of the Home-HF study. Eur J Heart Fail. 2009; 11(3): 319–25. DOI: 10.1093/eurjhf/hfn050 19174529 PMC2645059

[26] Kurtz B, Lemercier M, Pouchin SC, Benmokhtar E, Vallet C, Cribier A, et al. Automated home telephone self-monitoring reduces hospitalization in patients with advanced heart failure. J Telemed Telecare. 2011; 17(6): 298–302. DOI: 10.1258/jtt.2011.100901 21844176

[27] Melin M, Hägglund E, Ullman B, Persson H, Hagerman I. Effects of a tablet computer on self-care, quality of life, and knowledge: A randomized clinical trial. Journal of Cardiovascular Nursing. 2018; 33(4). DOI: 10.1097/JCN.0000000000000462 29369123

[28] Antonicelli R, Mazzanti I, Abbatecola AM, Parati G. Impact of home patient telemonitoring on use of β-blockers in congestive heart failure. Drugs Aging. 2010; 27(10): 801–5. DOI: 10.2165/11538210-000000000-00000 20883060

[29] Baker DW, Dewalt DA, Schillinger D, Hawk V, Ruo B, Bibbins-Domingo K, et al. The effect of progressive, reinforcing telephone education and counseling versus brief educational intervention on knowledge, self-care behaviors and heart failure symptoms. J Card Fail. 2011; 17(10): 789–96. DOI: 10.1016/j.cardfail.2011.06.374 21962415 PMC3185245

[30] Blum K, Gottlieb SS. The effect of a randomized trial of home telemonitoring on medical costs, 30-day readmissions, mortality, and health-related quality of life in a cohort of community-dwelling heart failure patients. J Card Fail. 2014; 20(7): 513–21. DOI: 10.1016/j.cardfail.2014.04.016 24769270

[31] DeWalt DA, Malone RM, Bryant ME, Kosnar MC, Corr KE, Rothman RL, et al. A heart failure self-management program for patients of all literacy levels: A randomized, controlled trial [ISRCTN11535170]. BMC Health Services Research. 2006; 6(1): 30. DOI: 10.1186/1472-6963-6-30 16533388 PMC1475568

[32] Mortara A, Pinna GD, Johnson P, Maestri R, Capomolla S, La Rovere MT, et al. Home telemonitoring in heart failure patients: the HHH study (Home or Hospital in Heart Failure). Eur J Heart Fail. 2009; 11(3): 312–8. DOI: 10.1093/eurjhf/hfp022 19228800 PMC2645060

[33] Seto E, Leonard KJ, Cafazzo JA, Barnsley J, Masino C, Ross HJ. Mobile phone-based telemonitoring for heart failure management: A randomized controlled trial. J Med Internet Res. 2012; 14(1): e31. DOI: 10.2196/jmir.1909 22356799 PMC3374537

[34] Wakefield BJ, Ward MM, Holman JE, Ray A, Scherubel M, Burns TL, et al. Evaluation of home telehealth following hospitalization for heart failure: A randomized trial. Telemed J E Health. 2008; 14(8): 753–61. DOI: 10.1089/tmj.2007.0131 18954244

[35] Woodend AK, Sherrard H, Fraser M, Stuewe L, Cheung T, Struthers C. Telehome monitoring in patients with cardiac disease who are at high risk of readmission. Heart Lung. 2008; 37(1): 36–45. DOI: 10.1016/j.hrtlng.2007.04.004 18206525

[36] Krum H, Forbes A, Yallop J, Driscoll A, Croucher J, Chan B, et al. Telephone support to rural and remote patients with heart failure: The Chronic Heart Failure Assessment by Telephone (CHAT) study. Cardiovasc Ther. 2013; 31(4): 230–7. DOI: 10.1111/1755-5922.12009 23061492

[37] Andrews E, Berghofer K, Long J, Prescott A, Caboral-Stevens M. Satisfaction with the use of telehealth during COVID-19: An integrative review. Int J Nurs Stud Adv. 2020; 2: 100008. DOI: 10.1016/j.ijnsa.2020.100008 33083791 PMC7564757

[38] Isautier JM, Copp T, Ayre J, Cvejic E, Meyerowitz-Katz G, Batcup C, et al. People’s experiences and satisfaction with telehealth during the COVID-19 pandemic in Australia: Cross-Sectional survey study. J Med Internet Res. 2020; 22(12): e24531. DOI: 10.2196/24531 33156806 PMC7732356

[39] Seferovic PM, Ponikowski P, Anker SD, Bauersachs J, Chioncel O, Cleland JGF, et al. Clinical practice update on heart failure 2019: Pharmacotherapy, procedures, devices and patient management. An expert consensus meeting report of the Heart Failure Association of the European Society of Cardiology. Eur J Heart Fail. 2019; 21(10): 1169–86. DOI: 10.1002/ejhf.1531 31129923

[40] Ahmed A, Aronow WS, Fleg JL. Higher New York Heart Association classes and increased mortality and hospitalization in patients with heart failure and preserved left ventricular function. Am Heart J. 2006; 151(2): 444–50. DOI: 10.1016/j.ahj.2005.03.066 16442912 PMC2771182

[41] Magsamen-Conrad K, Dillon JM, Billotte Verhoff C, Faulkner SL. Online health-information seeking among older populations: Family influences and the role of the medical professional. Health Commun. 2019; 34(8): 859–71. DOI: 10.1080/10410236.2018.1439265 29474125 PMC6230499

[42] Koehler F, Winkler S, Schieber M, Sechtem U, Stangl K, Böhm M, et al. Impact of remote telemedical management on mortality and hospitalizations in ambulatory patients with chronic heart failure. Circulation. 2011; 123(17): 1873–80. DOI: 10.1161/CIRCULATIONAHA.111.018473 21444883

[43] Organization WH. Implementing telemedicine services during COVID-19: Guiding principles and considerations for a stepwise approach. 2021.

[44] Frederix I, Caiani EG, Dendale P, Anker S, Bax J, Böhm A, et al. ESC e-Cardiology working group position paper: Overcoming challenges in digital health implementation in cardiovascular medicine. Eur J Prev Cardiol. 2019; 26(11): 1166–77. DOI: 10.1177/2047487319832394 30917695

[45] Klindtworth K, Oster P, Hager K, Krause O, Bleidorn J, Schneider N. Living with and dying from advanced heart failure: Understanding the needs of older patients at the end of life. BMC Geriatrics. 2015; 15(1): 125. DOI: 10.1186/s12877-015-0124-y 26470713 PMC4608315

[46] Henkel DM, Redfield MM, Weston SA, Gerber Y, Roger VL. Death in heart failure. Circulation: Heart Failure. 2008; 1(2): 91–7. DOI: 10.1161/CIRCHEARTFAILURE.107.743146 19300532 PMC2657718

[47] Lee DS, Gona P, Albano I, Larson MG, Benjamin EJ, Levy D, et al. A systematic assessment of causes of death after heart failure onset in the community: Impact of age at death, time period, and left ventricular systolic dysfunction. Circulation Heart Failure. 2011; 4(1): 36–43. DOI: 10.1161/CIRCHEARTFAILURE.110.957480 21071547 PMC3243964

[48] Italia L, Tomasoni D, Bisegna S, Pancaldi E, Stretti L, Adamo M, et al. COVID-19 and heart failure: From epidemiology during the pandemic to myocardial injury, myocarditis, and heart failure sequelae. Frontiers in cardiovascular medicine. 2021; 8. DOI: 10.3389/fcvm.2021.713560 PMC8382715 34447795

[49] Mosalpuria K, Agarwal SK, Yaemsiri S, Pierre-Louis B, Saba S, Alvarez R, et al. Outpatient management of heart failure in the United States, 2006–2008. Tex Heart Inst J. 2014; 41(3): 253–61. DOI: 10.14503/THIJ-12-2947 24955039 PMC4060338

[50] Lindsey BB, Villabona-Arenas CJ, Campbell F, Keeley AJ, Parker MD, Shah DR, et al. Characterising within-hospital SARS-CoV-2 transmission events using epidemiological and viral genomic data across two pandemic waves. Nature Communications. 2022; 13(1): 671. DOI: 10.1038/s41467-022-28291-y PMC8814040 35115517

[51] Tada A, Omote K, Nagai T, Honda Y, Nakano H, Honda S, et al. Prevalence, determinants, and prognostic significance of hospital acquired pneumonia in patients with acute heart failure. J Clin Med. 2020; 9(7): 2219. DOI: 10.3390/jcm9072219 32668753 PMC7408712

[52] Armaignac DL, Saxena A, Rubens M, Valle CA, Williams L-MS, Veledar E, et al. Impact of telemedicine on mortality, length of stay, and cost among patients in progressive care units: Experience from a large healthcare system. Crit Care Med. 2018; 46(5): 728–35. DOI: 10.1097/CCM.0000000000002994 29384782 PMC5908255

[53] Ekeland AG, Bowes A, Flottorp S. Effectiveness of telemedicine: A systematic review of reviews. Int J Med Inform. 2010; 79(11): 736–71. DOI: 10.1016/j.ijmedinf.2010.08.006 20884286

[54] Steventon A, Ariti C, Fisher E, Bardsley M. Effect of telehealth on hospital utilisation and mortality in routine clinical practice: a matched control cohort study in an early adopter site. BMJ Open. 2016; 6(2): e009221. DOI: 10.1136/bmjopen-2015-009221 PMC4746461 26842270

[55] Heo S, Lennie TA, Okoli C, Moser DK. Quality of life in patients with heart failure: Ask the patients. Heart & Lung: The Journal of Critical Care. 2009; 38(2): 100–8. DOI: 10.1016/j.hrtlng.2008.04.002 19254628 PMC2671196

[56] Juenger J, Schellberg D, Kraemer S, Haunstetter A, Zugck C, Herzog W, et al. Health related quality of life in patients with congestive heart failure: Comparison with other chronic diseases and relation to functional variables. Heart. 2002; 87(3): 235–41. DOI: 10.1136/heart.87.3.235 11847161 PMC1767036

[57] Pang L, Liu Z, Lin S, Liu Z, Liu H, Mai Z, et al. The effects of telemedicine on the quality of life of patients with lung cancer: A systematic review and meta-analysis. Ther Adv Chronic Dis. 2020; 11: 2040622320961597. DOI: 10.1177/2040622320961597 33101621 PMC7549184

[58] Cartwright M, Hirani SP, Rixon L, Beynon M, Doll H, Bower P, et al. Effect of telehealth on quality of life and psychological outcomes over 12 months (Whole Systems Demonstrator telehealth questionnaire study): Nested study of patient reported outcomes in a pragmatic, cluster randomised controlled trial. BMJ: British Medical Journal. 2013; 346: f653. DOI: 10.1136/bmj.f653 23444424 PMC3582704

[59] Salah HM, Minhas AMK, Khan MS, Pandey A, Michos ED, Mentz RJ, et al. Causes of hospitalization in the USA between 2005 and 2018. European Heart Journal Open. 2021; 1(1): oeab001. DOI: 10.1093/ehjopen/oeab001 35919090 PMC9242058

[60] Lahoz R, Fagan A, McSharry M, Proudfoot C, Corda S, Studer R. Recurrent heart failure hospitalizations are associated with increased cardiovascular mortality in patients with heart failure in Clinical Practice Research Datalink. ESC heart failure. 2020; 7(4): 1688–99. DOI: 10.1002/ehf2.12727 32383551 PMC7373936

[61] Fonarow GC, Abraham WT, Albert NM, Stough WG, Gheorghiade M, Greenberg BH, et al. Factors identified as precipitating hospital admissions for heart failure and clinical outcomes: Findings from OPTIMIZE-HF. Archives of Internal Medicine. 2008; 168(8): 847–54. DOI: 10.1001/archinte.168.8.847 18443260

[62] O’Connor M, Asdornwised U, Dempsey ML, Huffenberger A, Jost S, Flynn D, et al. Using telehealth to reduce all-cause 30-day hospital readmissions among heart failure patients receiving skilled home health services. Appl Clin Inform. 2016; 7(2): 238–47. DOI: 10.4338/ACI-2015-11-SOA-0157 27437037 PMC4941836

[63] Kotooka N, Kitakaze M, Nagashima K, Asaka M, Kinugasa Y, Nochioka K, et al. The first multicenter, randomized, controlled trial of home telemonitoring for Japanese patients with heart failure: Home telemonitoring study for patients with heart failure (HOMES-HF). Heart Vessels. 2018; 33(8): 866–76. DOI: 10.1007/s00380-018-1133-5 29450689

[64] Chaudhry SI, Mattera JA, Curtis JP, Spertus JA, Herrin J, Lin Z, et al. Telemonitoring in patients with heart failure. New England Journal of Medicine. 2010; 363(24): 2301–9. DOI: 10.1056/NEJMoa1010029 21080835 PMC3237394

[65] DeBusk RF, Miller NH, Parker KM, Bandura A, Kraemer HC, Cher DJ, et al. Care management for low-risk patients with heart failure. Annals of Internal Medicine. 2004; 141(8): 606–13. DOI: 10.7326/0003-4819-141-8-200410190-00008 15492340

[66] Xu H, Granger BB, Drake CD, Peterson ED, Dupre ME. Effectiveness of telemedicine visits in reducing 30-day readmissions among patients with heart failure during the COVID-19 pandemic. J Am Heart Assoc. 2022; 11(7): e023935. DOI: 10.1161/JAHA.121.023935 35229656 PMC9075458

[67] Rhatomy S, Prasetyo TE. Impact of COVID-19 on primary care visits: Lesson learnt from the early pandemic period. Journal of Community Empowerment for Health. 2020; 3(2). DOI: 10.22146/jcoemph.57918

[68] Suspensi (Penghentian Sementara) Layanan Kewajiban Pelayanan Universal/Universal Service Obligation (KPU/USO) [press release]. Jakarta: The Ministry of Communication and Information Republic of Indonesia2015.

[69] Rizkinaswara L. Penggunaan internet di Indonesia Indonesia: Kementerian Komunikasi dan Informatika RI; 2019 [Available from: https://aptika.kominfo.go.id/2019/08/penggunaan-internet-di-indonesia/.

[70] Human and Resources Department BK. Telemedicine jadi kunci layanan kesehatan di masa pandemi Indonesia: Badan Penyelanggara Jaminan Sosial; 2022 [Available from: https://www.bpjs-kesehatan.go.id/bpjs/post/read/2022/2320/Telemedicine-Jadi-Kunci-Layanan-Kesehatan-di-Masa-Pandemi.

[71] Indonesia MoHotRo. Blueprint of digital health transformation strategy. Indonesia: Ministry of Health of the Republic of Indonesia; 2021.

[72] Indonesia KKR. ASN Kemenkes Didorong Lahirkan Inovasi Baru Untuk Tingkatkan Pelayanan Kesehatan Indonesia: Kementrian Kesehatan Republik Indonesia; 2022.

[73] Dwiputra B. Harkit I-CARE Jakarta, Indonesia: Harkit I-CARE; 2022.

[74] Thomas S, Rich MW. Epidemiology, pathophysiology, and prognosis of heart failure in the elderly. Heart Fail Clin. 2007; 3(4): 381–7. DOI: 10.1016/j.hfc.2007.07.004 17905375 PMC5391148

[75] A S, AB A, RIP S, W S, AA S. Cost-Effectiveness of Telemedicine in Asia: A Scoping Review. Journal of Multidisciplinary Healthcare. 2021; 14: 3587–96. DOI: 10.2147/JMDH.S332579 35002248 PMC8721158

[76] Nguyen HV, Tan GS, Tapp RJ, Mital S, Ting DS, Wong HT, et al. Cost-effectiveness of a National Telemedicine Diabetic Retinopathy Screening Program in Singapore. Ophthalmology. 2016; 123(12): 2571–80. DOI: 10.1016/j.ophtha.2016.08.021 27726962

[77] Kruse CS, Soma M, Pulluri D, Nemali NT, Brooks M. The effectiveness of telemedicine in the management of chronic heart disease – A systematic review. JRSM Open. 2017; 8(3): 2054270416681747. DOI: 10.1177/2054270416681747 28321319 PMC5347273

[78] Vestergaard AS, Hansen L, Sørensen SS, Jensen MB, Ehlers LH. Is telehealthcare for heart failure patients cost-effective? An economic evaluation alongside the Danish TeleCare North heart failure trial. BMJ Open. 2020; 10(1): e031670. DOI: 10.1136/bmjopen-2019-031670 PMC7045102 31992604

[79] Tersalvi G, Winterton D, Cioffi GM, Ghidini S, Roberto M, Biasco L, et al. Telemedicine in heart failure during COVID-19: A step into the future. Frontiers in Cardiovascular Medicine. 2020; 7. DOI: 10.3389/fcvm.2020.612818 33363223 PMC7755592

[80] Silva-Cardoso J, Juanatey JRG, Comin-Colet J, Sousa JM, Cavalheiro A, Moreira E. The future of telemedicine in the management of heart failure patients. Card Fail Rev. 2021; 7: e11. DOI: 10.15420/cfr.2020.32 34136277 PMC8201465

[81] Organization WH. Telemedicine: Opportunities and developments in Member States: Report on the second global survey on eHealth 2009. Geneva: World Health Organization; 2010.

